# Normal spirometry equates to normal impulse oscillometry in healthy subjects

**DOI:** 10.1186/s12931-021-01693-0

**Published:** 2021-03-31

**Authors:** Brian Lipworth, Rory Chan

**Affiliations:** Scottish Centre for Respiratory Research, Ninewells Hospital, Dundee, DD19SY UK

## Background

Impulse oscillometry (IOS) is an effort independent test performed with tidal breathing which can ascertain the presence of small airways dysfunction by measuring the heterogeneity of resistance between 5 and 20 Hz (R5–R20) or the area under the reactance curve (AX). The article of Li et al. reported on impulse oscillometry (IOS) values in young non smoking symptomatic otherwise healthy subjects with preserved spirometry without a diagnosis of chronic respiratory disease [1]. In subjects who had forced expiratory flow rates < 65% predicted they reported poor area under curve values < 0.70 with associated low sensitivity and specificity values on the whole being less than 80%.

In our clinic we would consider pragmatic abnormal IOS values as R5 > 0.5 kPa/l.s, R5–R20 > 0.1 kPa/l/s and AX > 1.0 kPa/l, in terms of being indicative of a clinically relevant degree of airflow obstruction [2]. We would also like to point out that in persistent asthma patients with preserved spirometry who have FEV1 > 80% predicted, the presence of raised values for R5–R20 was associated with a greater likelihood of long term poor disease control [3]. Moreover small airways dysfunction as R5–R20 and AX is related to type 2 inflammation in asthma [4].

Li et al. have demonstrated that otherwise healthy individuals with symptoms who have abnormal forced expiratory flows also have normal IOS values. Moreover it is important to note that forced expiratory rates are rather volume dependent such that false positive low values are commonly seen when subjects do not exhale fully to residual volume. We would therefore have concerns that clinicians might end up erroneously interpreting what are essentially normal IOS values which would lead to unnecessary further investigation and cause potential unwarranted anxiety to patients with normal tests.

## Conclusions

Impulse oscillometry should be interpreted in the light of abnormal values as being indicative of small airways dysfunction.

## Data Availability

Not applicable.

## Abbreviations

R5: Resistance at 5 Hz (total resistance)
R20: Resistance at 20 Hz (central resistance)
R5–R20: Resistance heterogeneity between 5 and 20 Hz (peripheral resistance)
AX: Area under reactance curve
FEV1: Forced expiratory volume in 1 s
IOS: Impulse oscillometry
